# Use of artificial intelligence for gestational age estimation: a systematic review and meta-analysis

**DOI:** 10.3389/fgwh.2025.1447579

**Published:** 2025-01-30

**Authors:** Sabahat Naz, Sahir Noorani, Syed Ali Jaffar Zaidi, Abdu R. Rahman, Saima Sattar, Jai K. Das, Zahra Hoodbhoy

**Affiliations:** ^1^ Department of Pediatrics and Child Health, The Aga Khan University, Karachi, Pakistan; ^2^ Institute for Global Health and Development, The Aga Khan University, Karachi, Pakistan

**Keywords:** gestational age estimation, fetal ultrasound, artificial intelligence, accuracy, pregnancy

## Abstract

**Introduction:**

Estimating a reliable gestational age (GA) is essential in providing appropriate care during pregnancy. With advancements in data science, there are several publications on the use of artificial intelligence (AI) models to estimate GA using ultrasound (US) images. The aim of this meta-analysis is to assess the accuracy of AI models in assessing GA against US as the gold standard.

**Methods:**

A literature search was performed in PubMed, CINAHL, Wiley Cochrane Library, Scopus, and Web of Science databases. Studies that reported use of AI models for GA estimation with US as the reference standard were included. Risk of bias assessment was performed using Quality Assessment for Diagnostic Accuracy Studies-2 (QUADAS-2) tool. Mean error in GA was estimated using STATA version-17 and subgroup analysis on trimester of GA assessment, AI models, study design, and external validation was performed.

**Results:**

Out of the 1,039 studies screened, 17 were included in the review, and of these 10 studies were included in the meta-analysis. Five (29%) studies were from high-income countries (HICs), four (24%) from upper-middle-income countries (UMICs), one (6%) from low-and middle-income countries (LMIC), and the remaining seven studies (41%) used data across different income regions. The pooled mean error in GA estimation based on 2D images (*n* = 6) and blind sweep videos (*n* = 4) was 4.32 days (95% CI: 2.82, 5.83; *l*^2^: 97.95%) and 2.55 days (95% CI: −0.13, 5.23; *l*^2^: 100%), respectively. On subgroup analysis based on 2D images, the mean error in GA estimation in the first trimester was 7.00 days (95% CI: 6.08, 7.92), 2.35 days (95% CI: 1.03, 3.67) in the second, and 4.30 days (95% CI: 4.10, 4.50) in the third trimester. In studies using deep learning for 2D images, those employing CNN reported a mean error of 5.11 days (95% CI: 1.85, 8.37) in gestational age estimation, while one using DNN indicated a mean error of 5.39 days (95% CI: 5.10, 5.68). Most studies exhibited an unclear or low risk of bias in various domains, including patient selection, index test, reference standard, flow and timings and applicability domain.

**Conclusion:**

Preliminary experience with AI models shows good accuracy in estimating GA. This holds tremendous potential for pregnancy dating, especially in resource-poor settings where trained interpreters may be limited.

**Systematic Review Registration:**

PROSPERO, identifier (CRD42022319966).

## 1 Introduction

Optimal prenatal care relies on accurate gestational age (GA) estimation for appropriate care of mother and child during pregnancy and beyond (1). The precise dating of pregnancy is also necessary to assess viability in premature labor and post-date deliveries (1). The most common methods used to estimate GA during pregnancy are based on the last menstrual period (LMP) and ultrasonographic findings. Campbell et al. have reported that 45% of pregnant women are unsure of their LMP due to poor recall, irregular cycles, bleeding in early pregnancy, or oral contraceptive use within two months of conception (2). Appropriately performed prenatal ultrasound (US) has been shown to precisely estimate GA and is considered a gold standard (3, 4). According to the American College of Obstetricians and Gynecologists (ACOG), obstetric US in the first trimester is the most accurate method to confirm GA (5). For US, GA estimation requires the measurement of fetal parameters such as crown-rump length (CRL), head circumference (HC), abdominal circumference (AC), biparietal diameter (BPD), and femur length (FL) (5, 6). The error range in GA estimation in the first trimester is within one week, increasing to one to two weeks in the second trimester (5). US also provides real-time capability for the diagnosis of several conditions, such as multiple gestation, congenital anomalies, fetal growth restriction, and placental abnormalities (7, 8), and hence is recommended by the World Health Organization (WHO) at least once before the 24th week of gestation (9). Despite its advantages, access to the US is very poor in low-and middle-income countries (LMICs), mainly due to delays in access to prenatal care and lack of access to ultrasonographic machines and trained sonographers (10).

Over the past few years, increasing evidence has highlighted the use of artificial intelligence (AI) models in accurately analyzing image-based data in healthcare. AI in medicine aims to deal with disease prevention, diagnosis, and management in various fields, including pathology, oncology, radiology, etc. These models thus have the potential to meet the demand-supply issue by automating many image recognition tasks in medicine, thus reducing the required skill and resources (11).

Similar to other fields in medicine, which heavily rely on imaging, AI models have been used in multiple studies to measure major anatomical structures in the fetal US with reasonable accuracy (8, 12). It is thus important to synthesize the existing literature on the diagnostic accuracy of AI models for GA estimation to suggest the wide use of this technology, especially in resource-constrained settings.

## 2 Methods

This systematic review included studies that compared AI models against standard US measures. The systematic review protocol was registered on PROSPERO (CRD42022319966), and we followed the Preferred Reporting Items for Systematic Reviews and Meta-Analyses guidelines for reporting this publication (13).

### 2.1 Search strategy and data sources

A literature search was conducted on June 5, 2023, in PubMed Medline, the Cochrane Library, Scopus, and CINAHL. The search strategy used in PubMed was ((((“Artificial Intelligence”[Mesh] OR Artificial intelligence[tiab] OR AI OR “machine learning”[tiab] OR “deep learning”[tiab] OR “supervised learning”[tiab] OR “unsupervised learning”[tiab] OR “image recognition” OR “computer vision”)) AND ((“Ultrasonography”[Mesh] OR “Diagnostic Imaging”[Mesh] OR “Ultrasonography Doppler”[Mesh] OR Ultrasound OR doppler ultrasound))) AND ((“Pregnancy”[Mesh] OR pregnancy OR pregnant OR “pregnant women”[Mesh] OR “prenatal diagnosis”[Mesh] OR gestational age OR “fetal age” OR “Fetal Development”[Mesh] OR fetal assessment))) AND ((Accuracy OR diagnostic accuracy OR determination OR prediction OR estimation OR recall OR bias OR absolute error OR precision OR sensitivity OR specificity OR true positive OR false positive OR positive predictive value OR negative predictive value)). We manually searched bibliographies and citations of the included studies for cross-referencing that might have been overlooked during the literature search.

### 2.2 Eligibility criteria

We included all studies that reported the accuracy of an AI algorithm for the estimation of GA compared to the US as a reference standard. We included retrospective and prospective studies without any restriction on the trimester of enrolled pregnant women. Literature such as letters, opinions, commentaries, and narrative reviews were excluded. Also, those studies that used reference standards other than the US and published in a language besides English were excluded.

### 2.3 Data extraction

Two authors (ZH and SN) independently performed the screening of the titles and abstracts to determine potential eligibility. Two independent reviewers (SN and SNO) performed the full-text review and final decision on the studies to be included. Data was extracted in duplicate (SN and SNO) on pre-defined variables in an Excel sheet and extracted for baseline characteristics, year of publication, journal, title, aim/objectives, income region/setting, number of participants, study design, AI model used (refer to Supplementary Table S1), reference test, GA range for inclusion of participants, input measures, size of training and test set, validation method and study limitations. Any disagreements in inclusion or extraction were resolved by mutual discussion between the two reviewers (SN and SNO) or by contacting a third reviewer (ZH or JKD), and a decision was taken unanimously.

### 2.4 Risk of bias assessment

The risk of bias in the included studies was assessed by two independent authors (SN and SNO) using the Quality Assessment of Diagnostic Accuracy Studies-2 (QUADAS-2) tool (14). The risk of bias was assessed on four domains, i.e., patient selection, index test, reference standard, and flow and timing, with each of the domains included 3–4 specific questions. All studies included in this review were evaluated against these questions, and the responses were recorded as low-risk, high-risk, or unclear-risk for each question. If any question within a domain received a high-risk rating, the overall bias for that domain was deemed high.

In addition, the first three domains (patient selection, index test, and reference standard) were used to assess applicability concerns. For example, the patients included in the study did not match the review question, the conduct or interpretation of the index test was different from the review question, and the target condition defined by the reference standard did not match the review question.

### 2.5 Data analysis

The information from the studies was summarized in Table 1 based on study designs, sample size, size of the training and testing sets, and number of images used. The country where the study was performed was summarized according to their income regions by the World Bank (30). The performance metrics were summarized as mean errors in days, standard deviation (SD), 95% confidence intervals (CIs), accuracy, coefficient of determination (*R*^2^), and correlations (r). We performed meta-analysis (where possible) using mean absolute error (MAE) and conducted two different comparisons based on 2 dimensional (i.e., fetal head, fetal abdomen, femur) and blind sweep images. We also performed a sensitivity analysis of blind sweep videos after excluding one study with a high risk of bias. To standardize the reporting, we converted root-mean-squared error (RSME) into MAE (31). We also calculated SDs where CIs were available, and the SDs were then converted into standard errors (SEs) (32, 33).

**Table 1:** Summary of included studies.

Author & year	Country	Income region	Study design	Sample size	Input measures (2D images vs. blind sweeps)	Input measures (Video vs. Still)	Size of training & test set	GA range by gold standard (in weeks)	AI model used	Performance metrics (AI algorithm)	Validation method	External validation
Lee et al. (15)	USA, Zambia	HIC, MIC	Prospective cohort	3,842	Blind sweeps	Video	60% train & 20% test	CRL: 44–97 days, HC, AC, & FL: 98–195 days & 196–258 days	DNN	MAE (SD): Standard fetal biometry: 5.11 (4.7) Ensemble method: 3.6 (3.2) Video model: 3.63 (3.2) Image model: 3.97 (3.5)	60% train, 20% tune, and 20% test	No
Lee et al. (16)	Brazil, China, India, Italy, Kenya, Oman, UK USA, Pakistan, South Africa, Thailand	HIC, LMIC, UMIC	Prospective cohort	INTERGROWTH-21st (*n* = 4,233) INTERBIO-21st (*n* = 2,433)	HC, AC, & FL 2D images	Still	75% for training: 3,809 (219,974 images) & 10% for testing: 29,664 images	13 to 42 weeks	CNN	MAE Internal validation set: GA 13^+^^0^–42^+^^0^ weeks: +/− 3.5 GA 18^+^^0^–27^+^^6^ weeks: +/− 3.0 GA 28 ^+^^0^–42 ^+^^0^ weeks: +/− 4.3 External validation set: GA 13 ^+^^0^–42 ^+^^0^ weeks: +/− 4.1 GA 18 ^+^^0^–27 ^+^^6^ weeks: +/− 3.7 GA 28 ^+^^0^–42 ^+^^0^ weeks: +/− 5.0	1 dataset for training and internal validation, and 1 for external validation	Yes
Danet al. (17)	China	UMIC	Prospective cohort	7,113 women (10,413 images)	BPD, HC, AC, & FL 2D images	Still	Training set: 7,542 Test set: 1,832	2nd and 3rd trimesters	DNN (RESNET)	MAE (SD): 5.39 (4.01)	Internal validation: 134 and 74 External validation: 90	Yes
Arroyo et al. (18)	Peru	UMIC	Prospective pilot cohort study	58	Blind sweeps	Video	Training dataset: 30 & Hold-out Test Set: 28	3rd Trimester	DNN (U Net)	Mean (SD) U-Net: 225 (16.5) Standard of care: 223 (19.9)	80% training and 20% validatio	No
Alzubaidi et al. (19)	Netherlands	HIC	Prospective cohort	551	HC 2D images	Still	Training: 999 images Test: 335 images	14 to 40	DNN	MSE (r): 0.00072 (0.99)	80% training and 20% validation	No
Płotka et al. (20)	Poland	HIC	Prospective cohort	Dataset 1: 700 Dataset 2: 50 videos	HC, BPD, AC, & FL blind sweeps	Video	1st data set (80% training, 20% testing)	1st dataset: 15 to 38 weeks 2nd dataset: 19 to 38 weeks	CNN	MAE: 0.05 ± 0.01 week	Algorithm evaluated on 50 freehand fetal US video scans.	No
Pokaprakarn et al. (21)	USA & Zambia	HIC, MIC	Prospective cohort	4,521	Blind sweeps	Video	Training set: 3,509 (Training: 2,807, Tuning: 702) Test set: 1,012	9 to 37 weeks	DNN	MAE (+/−SE) 1st trimester: 2.1 +/− 0.19 days 2nd trimester: 3.1 +/− 0.16 days 3rd trimester 4.7 +/− 0.18 days	80% training, 20% tuning Test dataset: 1,012	No
Pei et al. (22)	China	UMIC	Retrospective	191 videos (29,829 2D images)	Gestational Sac 2D images	Still	NA	4·6–11	CNN	MAE: 1 +/− 0·76 weeks; 95% CI: 0·88, 1·12	NA	No
Prieto et al. (8)	Zambia, USA	HIC LMIC	Prospective Cohort	ZAPPS: 3,369 studies (23,209 images) UNC: 2,983 studies (124,646 images) FAMLI: 2,491 studies (7,233 images) Blind sweep	Blind Sweeps	Video	ZAPPS: 3,369 UNC: 2,983 & FAMLI: 2,491	ZAPPS: 13–18	DNN	MAE: 1·4 days	2 Sets for training & 1 for testing	Yes
Burgos-Artizzu et al. (23)	Spain	HIC	Prospective Cohort	3,386	Fetal brain (BPD & HC) AC & FL 2D images	Still	1,394 & 1,992	18–28 28–42 16–42	CNN	Avg error (CI error) (*R*^2^) 2·44 (6·7) (0·9) 5·49 (14·3) (0·91) 3·74 (11·0) (0·99)	41% data for training & 59% for testing	No
Fung et al. (24)	Brazil, China, India, Italy, Kenya, Oman, UK, USA	HIC LMIC UMIC	Prospective Cohort	Dataset 1: 4,607 Dataset 2: 3,067	HC, AC, & FL 2D images	Still	NA	20–30	Geometric ML Algorithm	Within 3 days	3 Groups for training & 1 for testing	Yes
Maraci et al. (12)	UK	HIC	Retrospective	Dataset A: 5,000 images Dataset B: 3,736 images	TCD 2D images	Still	Dataset A: 3,000 Dataset B: 500 & 3,236	16–26	CNN & FCN	Mean manual: 19·7 +/− 0·9 weeks Mean automated: 19·5 +/− 2·1 weeks	Dataset A: 3,000 images for training & 1,000 for validation Dataset B: 500 images for training & 3,236 for testing	Yes
van den Heuvel et al. (25)	Ethiopia	LMIC	Prospective Cohort	183	Blind Sweeps	Video	109 & 31	28–40	U-Net Architecture	MD: −3·6 days +/− 9·8	60% data for training, 20% for validation & 20% for testing	No
Papageorghiou et al. (26)	Brazil, China, India, Italy, Kenya, Oman, UK, USA	HIC LMIC UMIC	Prospective Cohort	4,229	HC, BPD, OFD, AC, & FL 2D images	Video	NA	14 26 >28	Genetic Algorithm	Mean Error (either direction): 6–7 days 12–14 days >14 days Adding FL improved model by 1–6 days across all trimesters whereas no improvement was reported by adding AC, BPD, OFD	NA	No
Namburete et al. (27)	Brazil, China, India, Italy, Kenya, Oman, UK, USA	HIC LMIC UMIC	Retrospective	157	Fetal brain (HC) 2D images	Still	447 & 187	18–27 +^6^ 28–33 +^6^ 18–33 +^6^	Regression forest Model	RMSE (CI) (r) 5·18 (10·10) (0·97) 7·77 (14·01) (0·83) 6·10 (11·64) (0·98)	NA	Yes
Fernández-Caballero et al. (28)	Spain	HIC	Retrospective	NA	BPD, AC, FL, & CRL 2D images	Still	NA	18	CNN (Region & Gradient-Based)	Mean = 17·6 weeks (using BPD) Mean = 18·2 weeks (using FL)	NA	No
Beksac et al. (29)	Turkey	UMIC	Prospective Cohort	143 (613 images)	HC & BPD 2D images	Still	552 & 61	14–38	ANN	In 98% of the cases, GA was estimated correctly	1 Set for training & 1 for testing	No

USA, United States of America; UK, United Kingdom; LMIC, low-and middle-income country; HIC, high-income country; UMIC, upper-middle income country; CNN, convolutional neural network; DNN, deep neural network; FCN, fully convolutional network; ML, machine learning; ANN, artificial neural network; ZAPPS, Zambian preterm birth prevention study; UNC, university of carolina maternal-fetal medicine group; FAMLI, fetal age machine learning initiative; HC, head circumference; BPD, biparietal diameter; AC, abdominal circumference; FL, femur length; CRL, crown-rump length; OFD, occipitofrontal diameter; TCD, trans cerebellar diameter; NA, not available; MRE, mean relative error; MAE, mean absolute error; CI, confidence interval; MD, mean difference; NICHD, national institute of child health and human development; RSME, root-mean-squared error.

Data was analyzed using STATA version 17 (34), where the pooled means with CIs were presented as forest plots. We assessed the heterogeneity using the *l*^2^ statistic, where the *l*^2^ value of >75% was considered higher heterogeneity. We reported random effect models in our review due to higher heterogeneity between studies. In addition, we conducted subgroup analyses for the comparison of 2D images only based on the study design (prospective/retrospective), pregnancy trimester, AI models, and external validation using the meta set command.

## 3 Results

**Figure 1:**
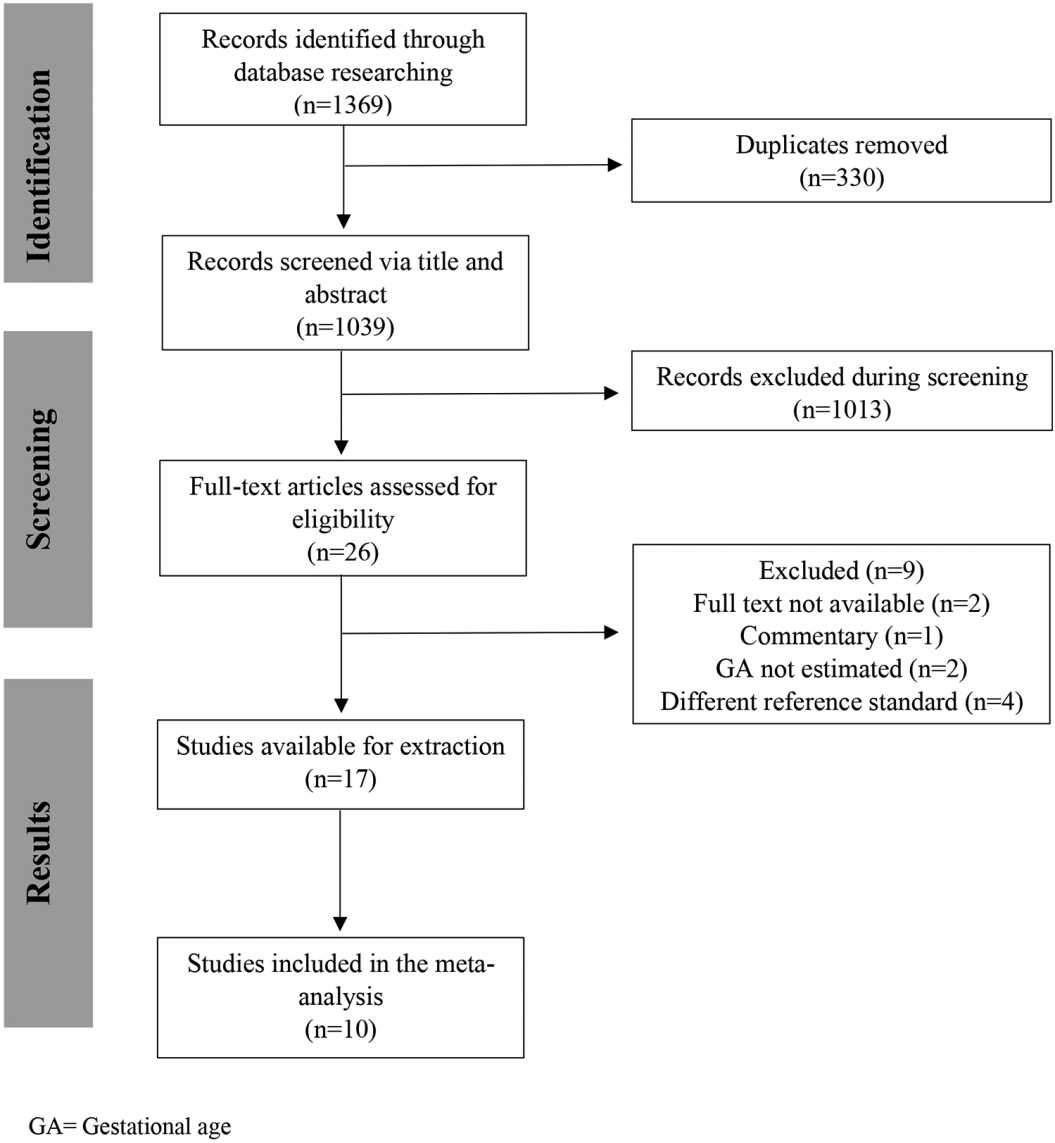
Search flow diagram.

The database search identified 1,039 studies after de-duplication; 17 met the eligibility criteria (8, 12, 15–29), and 10 studies were included in the meta-analysis (12, 15–18, 20–23, 27). The remaining seven studies (8, 19, 24–26, 28, 29) were not included as we were unable to get the information on mean errors in GA estimation, their standard deviation (SD) and/or confidence interval (CI) (Figure 1).

### 3.1 Characteristics of included studies

**Figure 2:**
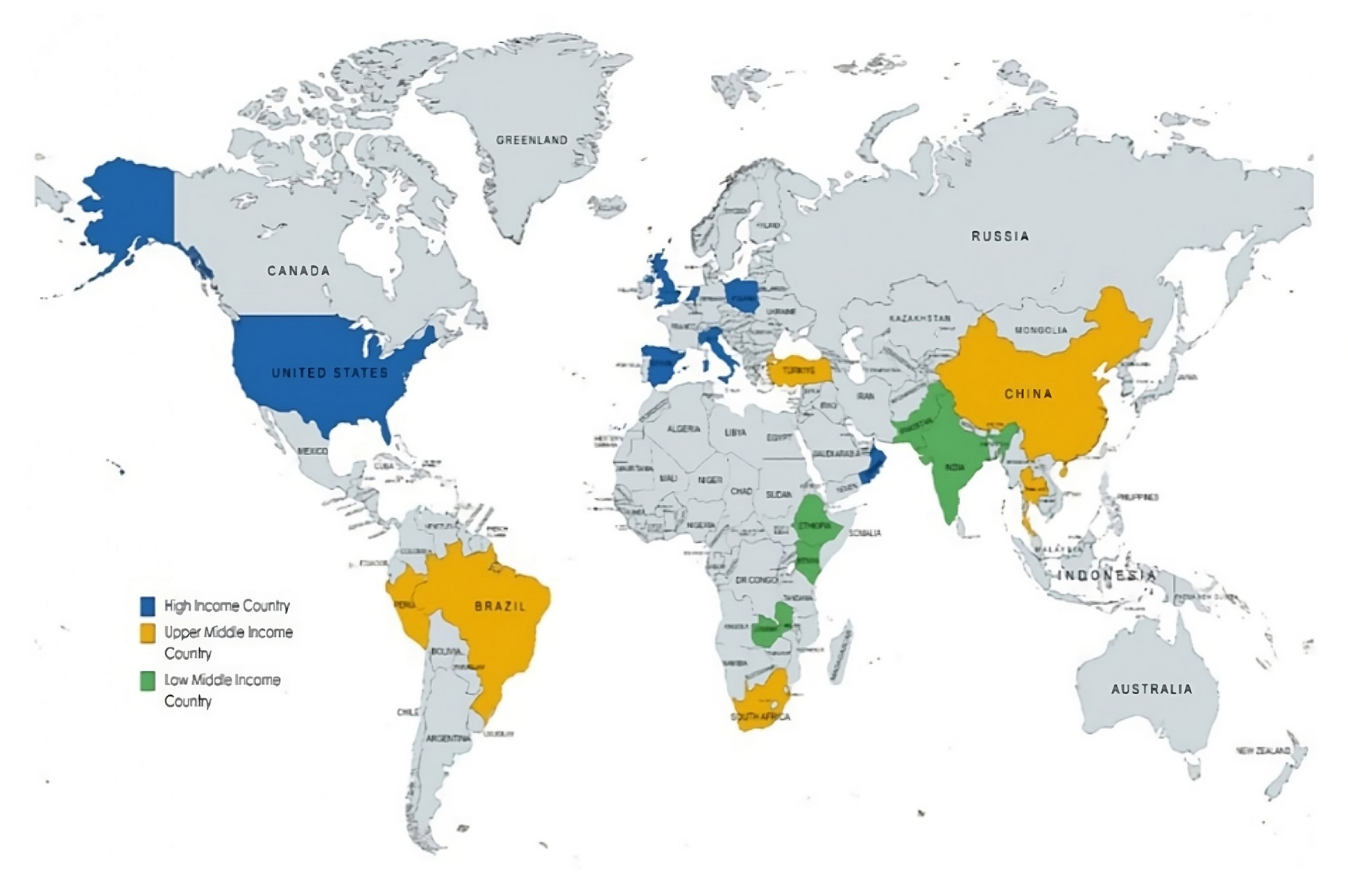
Distribution of countries according to income region contributed to the literature.

Among the 17 studies included in this review, five (29%) were conducted in high-income countries (HICs), four (24%) in upper-middle-income countries (UMICs), and one (6%) in low-and middle-income countries (LMIC). The remaining seven studies (41%) used data across different income regions, including HICs, UMICs, and LMICs (Figure 2).

The most common model used was neural network (*n* = 14) (8, 12, 15–23, 25, 28, 29), followed by regression forest model (27), geometric ML algorithm (24), and a genetic algorithm used by one study each (26). Thirteen studies (77%) (8, 15–21, 23–26, 29) were prospective cohorts, and four (23%) (12, 22, 27, 28) were retrospective studies. Most studies mentioned the number of participants ranging from 58 to 7,113 (8, 15–27, 29), while one study mentioned the number of images (5,000) (12), and one did not mention either (28). The size of training and test sets was described in 13 studies (8, 12, 15–21, 23, 25, 27, 29).

Eleven studies used 2D images (12, 16, 17, 19, 22–24, 26–29), while six used blind sweeps video (8, 15, 18, 20, 21, 25). While comparing the validation of the study findings, only six studies (35%) used external datasets to validate their results (8, 12, 16, 17, 24, 27) (Table 1).

### 3.2 Risk of bias assessment

**Figure 3:**
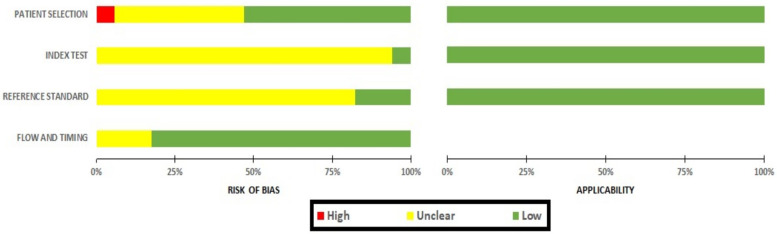
Risk of bias assessment and applicability concerns for included studies.

The risk of bias assessment for these studies has been presented in Figure 3. The bias on patient selection domain was low in nine studies (53%) (8, 12, 15–17, 21, 23, 24, 26), unclear in seven studies (43%) (19, 20, 22, 25, 27–29), whereas it was high in only one study (6%) (18).

In the majority of studies (*n* = 16; 94%) (12, 15–29), the risk of bias within the index domain was reported as unclear, with only one study showing low bias (*n* = 1; 6%) (8). Similarly, when assessing bias in the reference test, the majority of studies had unclear bias (*n* = 14; 82%) (12, 15–17, 19, 20, 22–24, 25–29), with the remaining three indicating low bias (*n* = 3; 18%) (8, 18, 21). This lack of clarity primarily stemmed from the absence of pre-specified thresholds in both the index and reference tests, as well as uncertain blinding statuses. The risk of bias for the flow and timing domain was low in 14 studies (82%) (8, 15, 16, 18–24, 26, 27, 29) and unclear in three studies (18%) (17, 25, 28). The risk of bias within the applicability domain, encompassing patient selection, the index test, and the reference standard, was consistently low in all the studies.

### 3.3 Outcome estimates

The GA range described in the included studies varied between four weeks and six days to 42 weeks. One study exclusively included scans from the first trimester (22), one from the second (12), and one from the third trimester (18). One study conducted separate analyses for the first, second, and third trimesters and combined these trimesters for the analysis (21). Three studies analyzed scans separately for the second and third trimesters and reported combined analysis for these (23, 27) while one study reported a combined analysis of all three trimesters (16). Two studies combined scans from the second and third trimesters (17, 20), and one reported results from all three combined (15).

**Figure 4:**
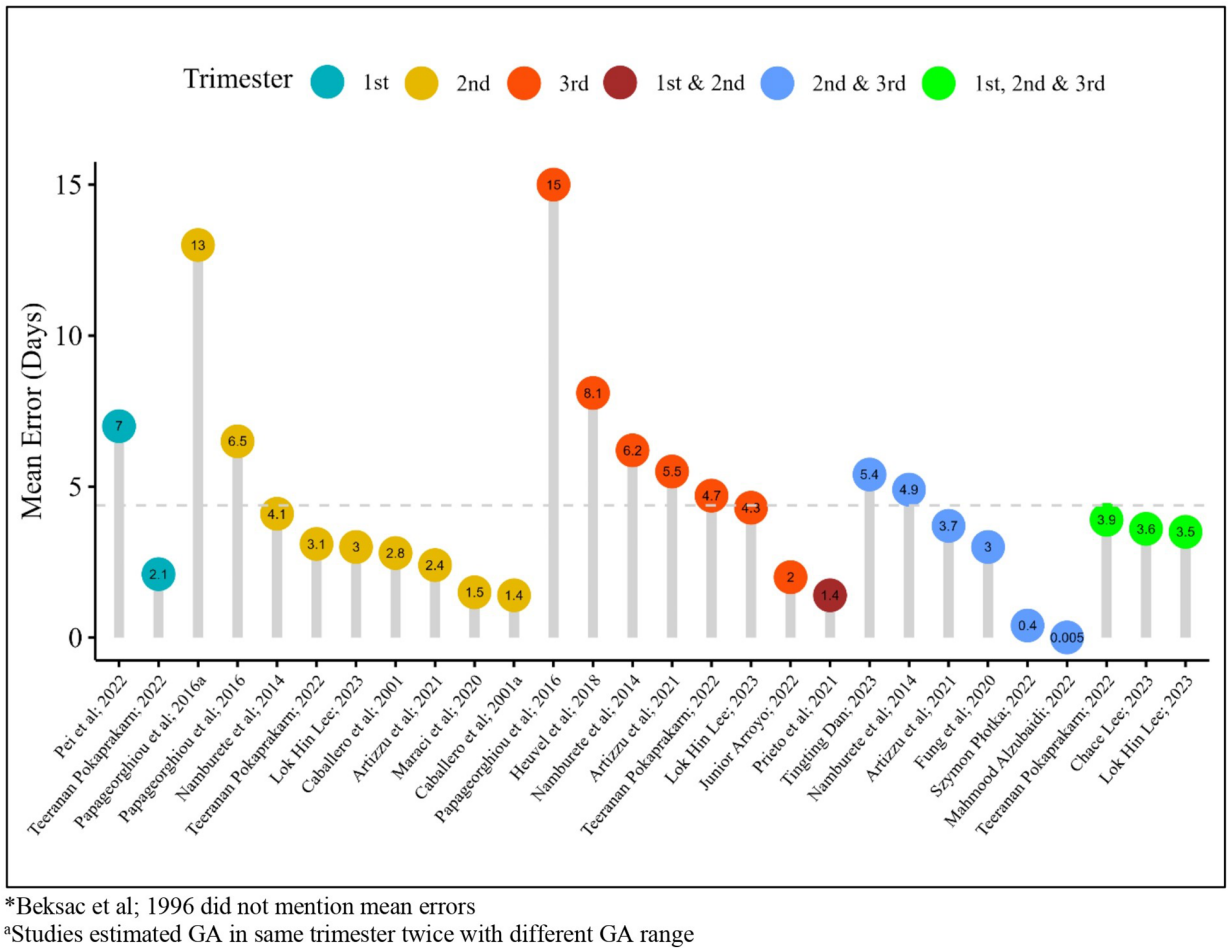
Mean errors in estimating gestational age across studies using AI.

The mean error in days in individual studies for GA estimation using AI models ranged between 0.005 (during the second and third trimesters combined) to 15 days (during the third trimester) throughout the trimesters across sixteen studies; one study did not mention mean errors (29) (Figure 4).

### 3.4 Overall estimates

**Figure 5:**
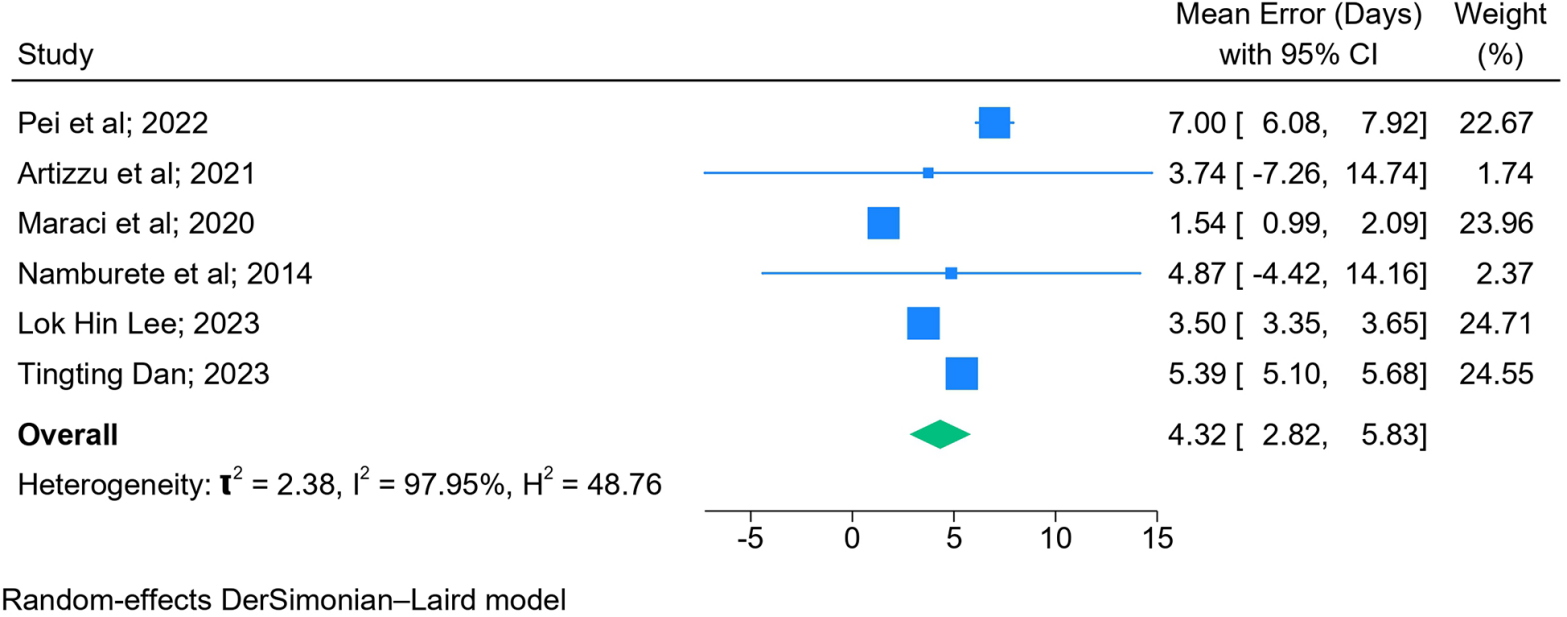
Forest plot of mean errors for overall GA estimation based on 2D images.

**Figure 6:**
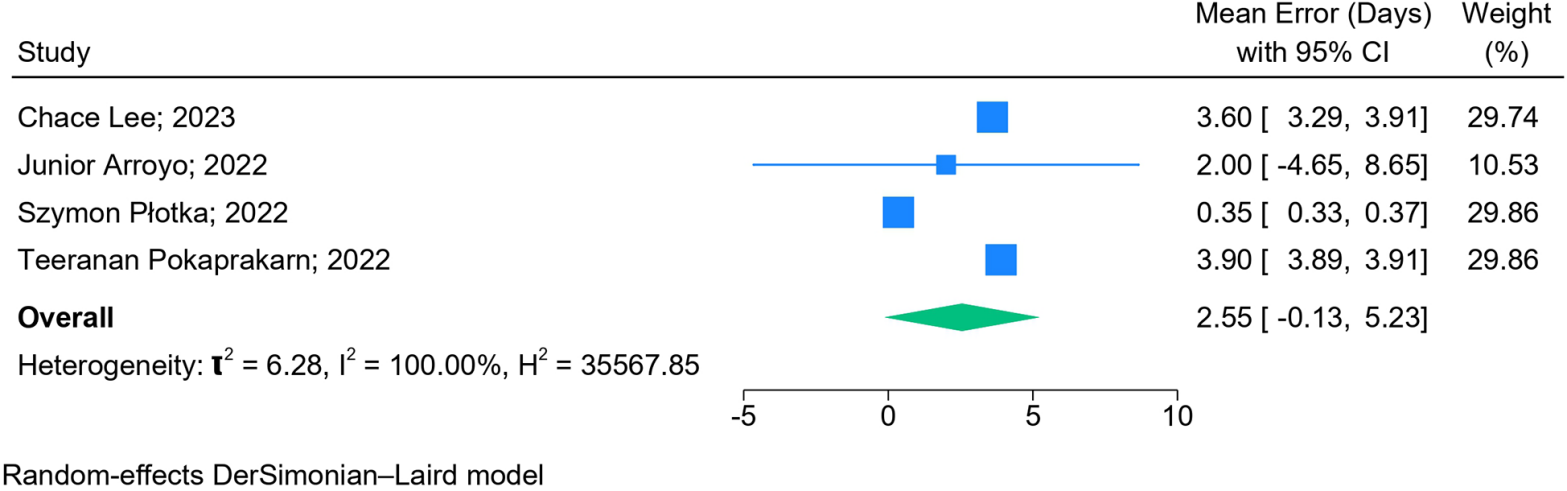
Forest plot of mean errors for overall GA estimation based on blind sweep videos.

The results of the meta-analysis based on 2D images (*n* = 6) (Figure 5) and blind sweep videos (*n* = 4) (Figure 6) suggested a pooled mean error of 4.32 days (95% CI: 2.82, 5.83; *l*^2^: 97.95%) and 2.55 days (95% CI: −0.13, 5.23; *l*^2^: 100%), respectively, in GA estimation across trimesters. The sensitivity analysis of blind sweep videos (*n* = 3) after excluding one study with a high risk of bias revealed a pooled mean error of 2.62 days (95% CI: −0.22, 5.45; *l*^2^: 100%) (Supplementary Figure S1).

### 3.5 Subgroup analyses based on trimesters (2D images)

The mean error in GA estimation during the first trimester was 7.00 days (95% CI: 6.08 7.92), 2.35 days (95% CI: 1.03, 3.67; *l*^2^: 87.97%) during the second, and 4.30 days (95% CI: 4.10, 4.50; *l*^2^: 0.00%) during the third trimester. The mean error in GA estimation was 5.39 days (95% CI: 5.10, 5.67; *l*^2^: 0.00%) during the second and third trimesters combined and 3.50 days (95% CI: 3.35, 3.65) during the first, second, and third trimesters combined (Supplementary Figure S2).

### 3.6 Subgroup analyses based on AI models (2D images)

Among the studies employing deep learning techniques, three of them with CNN found a mean error of 5.11 days (95% CI: 1.85, 8.37; *l*^2^: 96.31%) in GA estimation, while in one study used DNN, the reported mean error was 5.39 days (95% CI: 5.10, 5.68). In the remaining two studies, one study incorporated both CNN and FCN, resulting in a mean error of 1.54 days (95% CI: 0.99, 2.09), while the other study utilized a regression forest model and reported a mean error of 4.87 days (95% CI: −4.42, 14.16) in GA estimation (Supplementary Figure S3).

### 3.7 Subgroup analyses based on dataset validation (2D images)

The results of the meta-analysis based on external validation revealed that studies (*n* = 4) that used external datasets to validate their findings had a mean error of 3.73 days (CI: 2.36, 5.10; *l*^2^: 98.10%) in estimating GA as compared to those who internally validated their findings (5.11 days, CI: 1.85, 8.37; *l*^2^: 96.31%) (Supplementary Figure S4).

### 3.8 Subgroup analyses based on study designs (2D images)

Based on the study design used, three prospective studies reported a pooled mean error of 4.42 days (95% CI: 2.60, 6.24; *l*^2^: 98.48%), whereas the remaining three retrospective studies reported a pooled mean error of 4.36 days (95% CI: −0.48, 9.20; *l*^2^: 97.99%) in GA estimation (Supplementary Figure S5).

## 4 Discussion

This meta-analysis highlights the accuracy of AI models (mean difference of 4.32 days based on 2D images and 2.55 days based on blind sweep videos) in GA estimation using the US as the reference standard. It also highlights the accuracy of the various fetal biometric measures used to estimate GA in isolation and combination across the three trimesters.

Fetal US is a widely used modality for providing detailed information on fetal biometry to estimate accurate gestational age, improve diagnostic accuracy, and provide timely management (35). However, in LMICs, access to timely US, especially during the first trimester, is often challenging (10). In addition, the lack of skilled operators further compounds the issue, putting women and their newborns at risk of being undiagnosed and delivered without any opportunity to intervene (10). Similar to other radiological assessments where AI has helped bypass the need for a trained interpreter (36, 37), the current review demonstrates the promise offered by this technique in GA estimation, as evident from the findings of the meta-analysis. This review also demonstrates that using blind sweep images of the pregnant women's abdomen complemented by AI can be highly accurate in estimating GA. Although the number of studies with this technique is currently small (*n* = 4), this technology has immense potential to increase the reach of quality ultrasound to many marginalized communities. Blind sweeps also minimize the need for high quality images which may be difficult to obtain in communities where skilled personnel and high-end equipment are limited.

While comparing the sub-group analysis based on the GA, the estimates are more precise during the second (mean error: 2.35 days) compared to the first (mean error: 7.00 days) and third trimesters (mean error: 4.30 days). This variation in GA is very similar to when US-based GA is compared with pregnancies conceived through *in vitro* fertilization as a gold standard (approximately 1–3 days in the first and second trimester) (1). In addition, this variation is clinically acceptable as the impact of a small difference in GA (<7 days), may not have a sizeable impact on the estimation of preterm pregnancies. However, in the current study, there is only one study reported from the first trimester and hence precludes any further interpretation. This area is a gap in the literature as the ACOG guidelines suggest performing obstetric US during the first trimester as the most accurate method for GA estimation (5). Even though the second-trimester scans accurately estimate GA using AI models, we were unable to extract when the scan was performed. This would be important as scans done in the earlier half of the second trimester are more accurate than in the later half (5).

Various fetal biometric measurements used as 2D images (BPD, HC, AC, and FL) have been used manually in different combinations to estimate GA through various formulas (38). We found precise estimates (mean error: 2.98 days) in GA estimation, while all fetal biometrics (BPD, HC, FL, and AC) were included in the model. We also found good estimates for TCD (mean error: 1.54 days) and BPD and HC combined (mean error: 2.00 days); however, these were only reported by one study each.

Based on the AI models used, deep learning techniques such as CNN and DNN performed better than the regression model which was reported by only one study. Deep learning algorithms have been shown to perform on large image-based datasets (39). These algorithms are known to perform superior to conventional machine learning (ML) methods, especially when interacting with complex data (40). The current review has shown that using these algorithms, the GA variation is approximately 4 days, while that shown by actual ultrasound measurements ranges from 6 to 14 days across the trimesters (25). In the current use case of GA estimation, a number of fetal biometric parameters are measured, thus making the data set multi-dimensional and explaining the better performance of deep learning algorithms in estimating GA.

This review also highlights the need for standardized reporting of studies using AI on healthcare data. As shown in this review, there was significant heterogeneity in the performance metrics used to report GA along with other study parameters such as sample size, GA range, and input parameters. Furthermore, most of the studies in this review had unclear risk of bias for patient selection as information on the random selection of patients was not provided, as well as on the domain for index and reference tests due to unclarity of pre-defined thresholds or the blinding status of the interpreters/operators.

The existing literature on estimating GA using AI is mainly based on studies conducted in HICs and UMICs, whereas the evidence from LMICs is sparse. With preterm birth and its associated morbidities being the leading cause of neonatal deaths worldwide with a disproportionately high burden in LMICs (41), timely estimation of GA in pregnancy is important so that care pathways for women at high risk of preterm birth can be prioritized. Lack of a specialized health workforce and diagnostic infrastructure curtail the large-scale use of the US; however, technologies such as blind sweep images coupled with AI can help address the inequity in health services in these regions (42). To overcome these disparities between regions, establishing global collaborations may be a good approach to utilize the skills available in the Global North on datasets from the Global South thus reducing the equity gap. However, understanding the limitations of AI specifically in terms of its applicability in healthcare as these models rely on the availability of high-quality imaging, which may be challenging in resource-constraint settings. In addition, there is a significant demand for diverse and representative training datasets to ensure the models perform equitably across various demographic conditions. Moreover, ethical concerns, particularly issues related to data privacy, further complicate the integration of AI in healthcare.

To the best of our knowledge, this is the first meta-analysis on the use of AI in estimating GA. Furthermore, this review informs the need for further research in this area, especially from LMICs, along with the need for standardized reporting of the evaluation and external validation of the AI models. However, this study has several limitations. Out of the 17 studies included in this review, we could perform a meta-analysis only on ten studies due to the variability in the performance metrics reported across studies. In addition, the high heterogeneity observed between the included studies necessitates a cautious interpretation of the pooled estimates, even with the application of random-effects models. Even though there is a growing body of AI literature across the globe, we only included studies from the published literature in English, which could increase the risk of publication bias.

## 5 Conclusion

This review provides evidence on the performance of AI models to estimate GA using US as a gold standard. The findings from this review may be particularly relevant in LMICs, where there is a dearth of trained care providers and access to these is limited. Future research requires a standardized approach for assessing and reporting AI accuracy in GA estimation and inclusion of studies conducted in LMICs. Widescale implementation of AI algorithms in these regions would empower frontline care providers such as midwives for better decision-making during pregnancy, thus improving pregnancy outcomes.

## Data Availability

The data analyzed in this study is subject to the following licenses/restrictions: As a meta-analysis, this study used data that had already been reported in the primary literature. Requests for the derived data used to construct the meta-estimates are available on request by contacting the corresponding author. Requests to access these datasets should be directed to Zahra Hoodbhoy, zahra.hoodbhoy@aku.edu.
