# Supplementary material for: Use of artificial intelligence for gestational age estimation: a systematic review and meta-analysis
Source: Front Glob Womens Health. 2025 Jan 30;6:1447579. doi: 10.3389/fgwh.2025.1447579 (PMC11821921; doi:10.3389/fgwh.2025.1447579)
Supplement: Supplementary file 1 [file Datasheet1.pdf]

Use of artificial intelligence for gestational age estimation: A systematic review and meta-analysis	
**Comments from Reviewer 2**	**Responses**
This systematic review and meta-analysis provides a comprehensive and rigorous examination of the accuracy of AI models in estimating GA from ultrasound images. Conducted following the PRISMA-DTA guidelines and employing the QUADAS-2 tool for bias assessment, the study ensures methodological reliability and scientific rigor. The findings reveal that AI models achieve high accuracy in GA estimation, offering robust evidence of their potential to revolutionize prenatal care. This research is particularly significant in resource-limited settings, where trained personnel for interpreting ultrasound images are often scarce, underscoring the transformative role AI could play in improving maternal and fetal health outcomes in underserved regions.	Thank you for your detailed review and valuable feedback. We have incorporated the suggested revisions.
There are several aspects that could be further refined to enhance its overall impact and applicability.<br>The exclusion of non-English studies during the inclusion phase may have restricted the representation of data from low- and middle-income countries (LMICs). Incorporating studies from these regions would enhance the generalizability of the findings and shed light on the unique challenges and opportunities associated with implementing AI models in resource-constrained environments.	We agree with the reviewer that there could be some potential publication bias as we have reviewed only the studies that have been published in English language. However, since none of the team members are fluent in other languages, this undertaking would not have been feasible. We have mentioned this as a limitation on page 15 lines 342-344.
Furthermore, a more comprehensive discussion of the limitations of AI models is essential. Key considerations include their reliance on high-quality imaging, the significant demand for diverse and representative training datasets, and the ethical issues surrounding their application in healthcare. Addressing these factors would offer a more balanced and nuanced perspective on the current capabilities and constraints of AI in this context.	Thank you for your comment.<br>We have included the discussion on the limitations of AI models in terms of its applicability in healthcare on page 14, lines 329-334.
