# Supplementary material for: Use of artificial intelligence for gestational age estimation: a systematic review and meta-analysis
Source: Front Glob Womens Health. 2025 Jan 30;6:1447579. doi: 10.3389/fgwh.2025.1447579 (PMC11821921; doi:10.3389/fgwh.2025.1447579)
Supplement: Supplementary file 2 [file Datasheet2.pdf]

**Use of artificial intelligence for gestational age estimation: A systematic review and meta-analysis**	
**Comments from Reviewer 1**	**Responses**
I love the topic of this paper and think it is worth doing. I do not think it is a particularly useful analysis because there are two fundamentally different methods that are being combined into a single measure of accuracy. Here is what I mean:<br>1. Some of the papers describe the use of AI/deep learning to estimated GA directly from image features (i.e. Popaprakarn et al, Lee et al)<br>2. Other papers use AI to identify standard vies (head, abdomen and femur), measure those views and put those measurements into standard formulas such as Hadlock. In these cases, the AI algorithm is simply a workflow assistant rather than making a direct estimate of GA	We would like to thank the reviewer for their very valuable feedback on this important differentiation of estimating GA. We have added the type of imput measures (2D images vs blind sweeps) in Table 1 which describes the included studies.<br><br>We have also split the meta-analysis into two groups – one group for studies which used 2D images (n=6) and theother one where they used blind sweeps (n=4). Due to the small number of studies using blind sweeps, the sub group analysis on trimesters, study design, AI models used and external validation has been restricted to the 6 studies using 2D images.<br>These changes are present in the abstract on pages 2-3, methods section on pages 7-8, results section on pages 9-12 and discussion section on pages 12-14.
There are 2 papers that I know of that were not included in the metaanalysis and should be. These are Vishwanathan et al and Stringer et al both of which use the first approach (direct inference from the image and no formula used).	Thank you for highlighting these studies. For this review, we only included studies till June 2023. Hence these studies have not beein included in the current analysis.
The distinction the authors make between CNN and DNN is not helpful. It would be more useful for the reader to summarize the approaches as I have done above. Also described which ones used still frames vs videos would be useful.	Thank you for your comment. We have added the type of image (still vs video) in Table 1 of included studies. The analysis has also been revised as per comment 1.
