# Supplementary material for: Use of artificial intelligence for gestational age estimation: a systematic review and meta-analysis
Source: Front Glob Womens Health. 2025 Jan 30;6:1447579. doi: 10.3389/fgwh.2025.1447579 (PMC11821921; doi:10.3389/fgwh.2025.1447579)
Supplement: Supplementary file 3 [file Datasheet3.pdf]

Table 1: Summary of included studies

Author & Year	Country	Income Region	Study Design	Sample Size	Input Measures (2D images vs blind sweeps)	Input Measures (Video vs Still)	Size of Training & Test Set	GA Range by Gold Standard (in weeks)	AI model used	Performance Metrics (AI algorithm)	Validation Method	External Validation
Chace Lee, 2023	USA, Zambia	HIC, MIC	Prospective cohort	3842	Blind sweeps	Video	60% train & 20% test	CRL: 44-97 days, HC, AC, & FL: 98-195 days & 196-258 days	DNN	MAE (SD): Standard fetal biometry: 5.11 (4.7) Ensemble method: 3.6 (3.2) Video model: 3.63 (3.2) Image model: 3.97 (3.5)	60% train, 20% tune, and 20% test	No
Lok Hin Lee, 2023	Brazil, China, India, Italy, Kenya, Oman, UK USA, Pakistan, South Africa, Thailand	HIC, LMIC, UMIC	Prospective cohort	INTERGROWTH-21st (n =4233) INTERBIO-21st (n=2433)	HC, AC, & FL 2D images	Still	75% for training: 3809 (219,974 images) & 10% for testing: 29,664 images	13 to 42 weeks	CNN	MAE Internal validation set: GA 13^{+0}-42^{+0} weeks: +/- 3.5 GA 18^{+0}-27^{+6} weeks: +/- 3.0 GA 28^{+0}-42^{+0} weeks: +/- 4.3 External validation set: GA 13^{+0}-42^{+0} weeks: +/- 4.1 GA 18^{+0}-27^{+6} weeks: +/- 3.7 GA 28^{+0}-42^{+0} weeks: +/- 5.0	1 dataset for training and internal validation, and 1 for external validation	Yes
Tingting Dan,2023	China	UMIC	Prospective cohort	7113 women (10,413 images)	BPD, HC, AC, & FL 2D images	Still	Training set: 7542 Test set: 1832	2nd and 3rd trimesters	DNN (RESNET)	MAE (SD): 5.39 (4.01)	Internal validation: 134 and 74 External validation: 90	Yes
Junior Arroyo, 2022	Peru	UMIC	Prospective pilot cohort study	58	Blind sweeps	Video	Training dataset: 30 & Hold-out Test Set: 28	3rd Trimester	DNN (U Net)	Mean (SD) U-Net: 225 (16.5) Standard of care: 223 (19.9)	80% training and 20% validation	No
Mahmood Alzubaidi, 2022	Netherlands	HIC	Prospective cohort	551	HC 2D images	Still	Training: 999 images Test: 335 images	14 to 40	DNN	MSE (r): 0.00072 (0.99)	80% training and 20% validation	No
Szymon Płotka, 2022	Poland	HIC	Prospective cohort	Dataset 1: 700 Dataset 2: 50 videos	HC, BPD, AC, & FL blind sweeps	Video	1st data set (80% training, 20% testing)	1st dataset: 15 to 38 weeks 2nd dataset: 19 to 38 weeks	CNN	MAE: 0.05 ± 0.01 week	Algorithm evaluated on 50 freehand fetal US video scans.	No
Teeranan Pokaprakarn,2022	USA & Zambia	HIC, MIC	Prospective cohort	4521	Blind sweeps	Video	Training set: 3509 (Training: 2807, Tuning: 702) Test set: 1012	9 to 37 weeks	DNN	MAE (+/-SE) 1st trimester: 2.1 +/- 0.19 days 2nd trimester: 3.1 +/- 0.16 days 3rd trimester 4.7 +/- 0.18 days	80% training, 20% tuning Test dataset: 1012	No

Pei et al; 2022	China	UMIC	Retrospective	191 videos (29,829 2D images)	Gestational Sac 2D images	Still	NA	4·6-11	CNN	MAE: 1 +/- 0·76 weeks;<br>95% CI: 0·88, 1·12	NA	No
Prieto et al; 2021	Zambia, USA	HIC<br>LMIC	Prospective Cohort	ZAPPS: 3369 studies (23,209 images)<br>UNC: 2983 studies (124,646 images)<br>FAMLI: 2491 studies (7,233 images)<br>Blind sweep	Blind Sweeps	Video	ZAPPS: 3369<br>UNC: 2983 &<br>FAMLI: 2491	ZAPPS: 13-18	DNN	MAE: 1·4 days	2 Sets for training & 1 for testing	Yes
Artizzu et al; 2021	Spain	HIC	Prospective Cohort	3386	Fetal brain (BPD & HC) AC & FL 2D images	Still	1394 & 1992	 18-28<br>28-42<br>16-42	CNN	Avg error (CI error) (R^2)<br>2·44 (6·7) (0·9)<br>5·49 (14·3) (0·91)<br>3·74 (11·0) (0·99)	41% data for training & 59% for testing	No
Fung et al; 2020	Brazil, China, India, Italy, Kenya, Oman, UK, USA	HIC<br>LMIC<br>UMIC	Prospective Cohort	Dataset 1: 4607<br>Dataset 2: 3067	HC, AC, & FL 2D images	Still	NA	20-30	Geometric ML Algorithm	Within 3 days	3 Groups for training & 1 for testing	Yes
Maraci et al; 2020	UK	HIC	Retrospective	Dataset A: 5000 images<br>Dataset B: 3736 images	TCD 2D images	Still	Dataset A: 3000<br>Dataset B: 500 & 3236	16-26	CNN & FCN	Mean manual: 19·7 +/- 0·9 weeks<br>Mean automated: 19·5 +/- 2·1 weeks	Dataset A: 3000 images for training & 1000 for validation<br>Dataset B: 500 images for training & 3236 for testing	Yes
Heuvel et al; 2018	Ethiopia	LMIC	Prospective Cohort	183	Blind Sweeps	Video	109 & 31	28-40	U-Net Architecture	MD: -3·6 days +/- 9·8	60% data for training, 20% for validation & 20% for testing	No
Papageorghiou et al; 2016	Brazil, China, India, Italy, Kenya, Oman, UK, USA	HIC<br>LMIC<br>UMIC	Prospective Cohort	4229	HC, BPD, OFD, AC, & FL 2D images	Video	NA	 <br>14<br>26<br>>28	Genetic Algorithm	Mean Error (either direction):<br>6-7 days<br>12-14 days<br>> 14 days<br>Adding FL improved model by 1-6 days across all trimesters whereas no improvement was reported by adding AC, BPD, OFD	NA	No
Namburete et al; 2014	Brazil, China, India, Italy, Kenya,	HIC<br>LMIC<br>UMIC	Retrospective	157	Fetal brain (HC) 2D images	Still	447 & 187	 $18\text{-}27^{+6}$<br>$28\text{-}33^{+6}$<br>$18\text{-}33^{+6}$	Regression forest Model	RMSE (CI) (r)<br>5·18 (10·10) (0·97)<br>7·77 (14·01) (0·83)<br>6·10 (11·64) (0·98)	NA	Yes

	Oman, UK, USA											
Caballero et al; 2001	Spain	HIC	Retrospective	NA	BPD, AC, FL, & CRL 2D images	Still	NA	18	CNN (Region & Gradient-Based)	Mean = 17·6 weeks (using BPD)<br>Mean = 18·2 weeks (using FL)	NA	No
Beksac et al; 1996	Turkey	UMIC	Prospective Cohort	143 (613 images)	HC & BPD 2D images	Still	552 & 61	14-38	ANN	In 98% of the cases, GA was estimated correctly	1 Set for training & 1 for testing	No

USA= United States of America, UK= United Kingdom, LMIC= Low-and middle-income country, HIC= High-income country, UMIC= Upper-middle income country, CNN= Convolutional Neural Network, DNN= Deep Neural Network, FCN= Fully Convolutional Network, ML= Machine Learning, ANN= Artificial Neural Network, ZAPPS= Zambian Preterm Birth Prevention Study, UNC= University of Carolina Maternal-Fetal Medicine group, FAMLI= Fetal Age Machine Learning Initiative, HC= Head Circumference, BPD= Biparietal Diameter, AC= Abdominal Circumference, FL= Femur Length, CRL= Crown-Rump Length, OFD= Occipitofrontal Diameter, TCD= Trans Cerebellar Diameter, NA= Not available, MRE= Mean Relative Error, MAE: Mean Absolute Error, CI= Confidence Interval, MD= Mean Difference, NICHD= National Institute of Child Health and Human Development, RSME= Root-mean-squared error
