# Supplementary material for: Use of artificial intelligence for gestational age estimation: a systematic review and meta-analysis
Source: Front Glob Womens Health. 2025 Jan 30;6:1447579. doi: 10.3389/fgwh.2025.1447579 (PMC11821921; doi:10.3389/fgwh.2025.1447579)

Supplementary Table 1: Brief description of ML models:

Algorithm category	Description
**Neural Network [1]**	Mimics the biological neural network residing within the human brain to analyze data
**Deep Learning [2]**	Uses a combination of artificial neural networks in a computationally efficient manner
**Geometric ML algorithm [3]**	Geometric ML is a field of machine learning that can learn from complex data like graphs and multi-dimensional points. It seeks to apply traditional Convolutional Neural Networks to 3D objects, graphs, and manifolds
**Region-based Convolutional Neural Networks (R-CNN) [4]**	R-CNN are a family of machine learning models for computer vision and specifically object detection
**Genetic algorithm [5]**	A genetic algorithm is a search-based algorithm used for solving optimization problems in machine learning
**Ensemble learning [6]**	Ensemble Learning combines multiple base models to create a stronger, more robust model than its individual components. This approach leverages the diversity of base models to reduce the risk of overfitting and enhance predictive performance.

References:

1. *What are neural networks?* 2021.
2. Y.-S. Park, S.L., *Chapter 7 - Artificial Neural Networks: Multilayer Perceptron for Ecological Modeling*. Developments in Environmental Modelling. 2016.
3. Taylor, M. *Geometric deep learning: Convolutional Neural Networks on Graphs and Manifolds*. 2019.
4. Brownlee, J. *A Gentle Introduction to Object Recognition With Deep Learning*. 2019.
5. Muthee, A. *The Basics of Genetic Algorithms in Machine Learning*. 2021.
6. Kumar, V., P.S.S. Aydav, and S. Minz, *Multi-view ensemble learning using multi-objective particle swarm optimization for high dimensional data classification.* Journal of King Saud University-Computer and Information Sciences, 2022. **34**(10): p. 8523-8537.

Supplementary Figure 1: Forest plot of sensitivity analysis of mean errors for overall GA estimation based on blind sweep videos

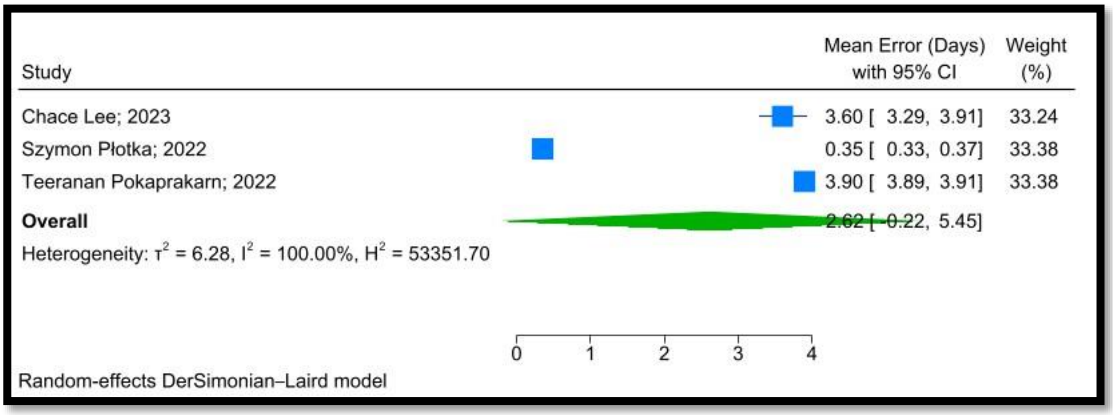

Supplementary Figure 2: Forest plot of mean errors in GA estimation based on trimesters

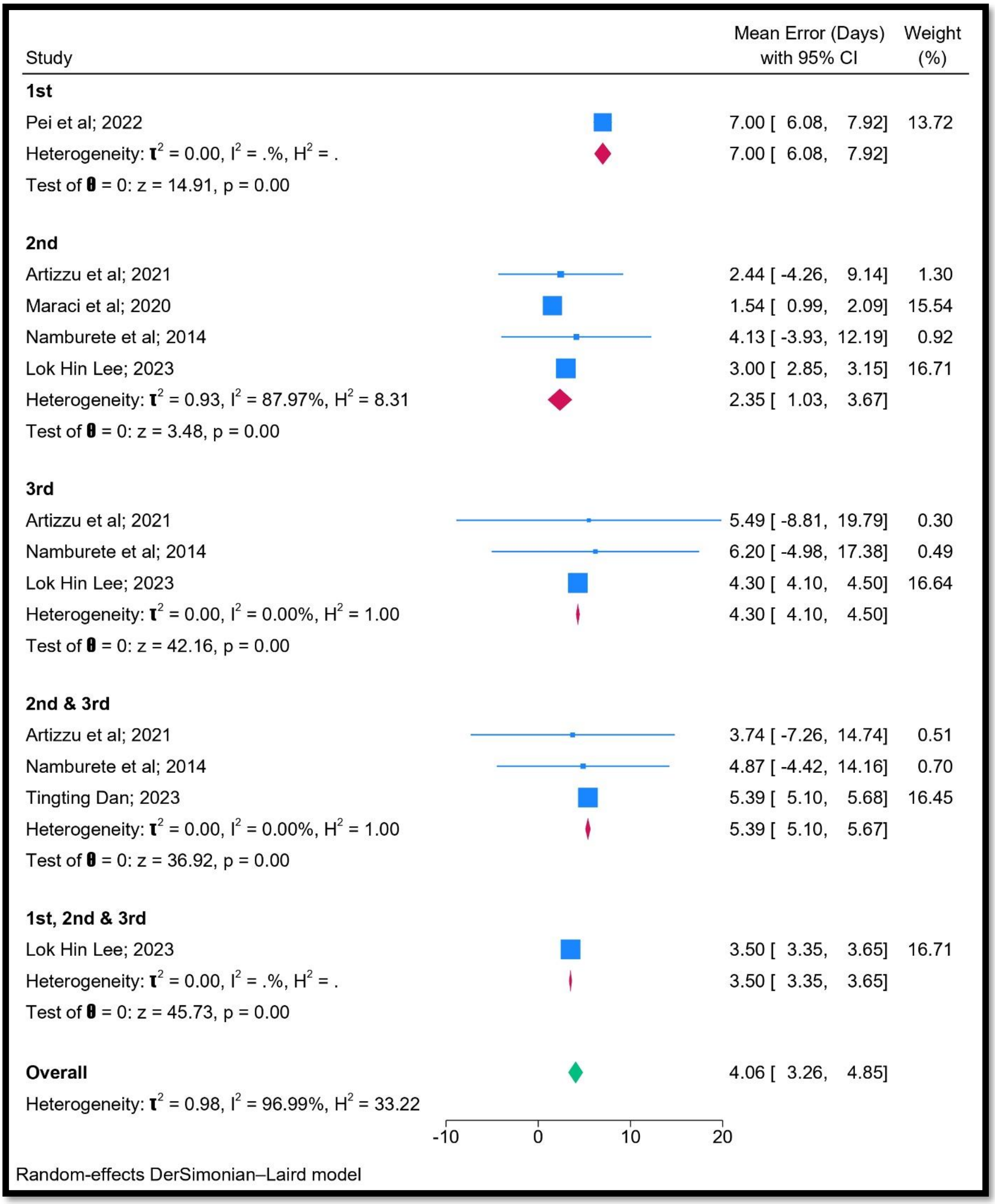

Supplementary Figure 3: Forest plot of mean errors in GA estimation based on AI models

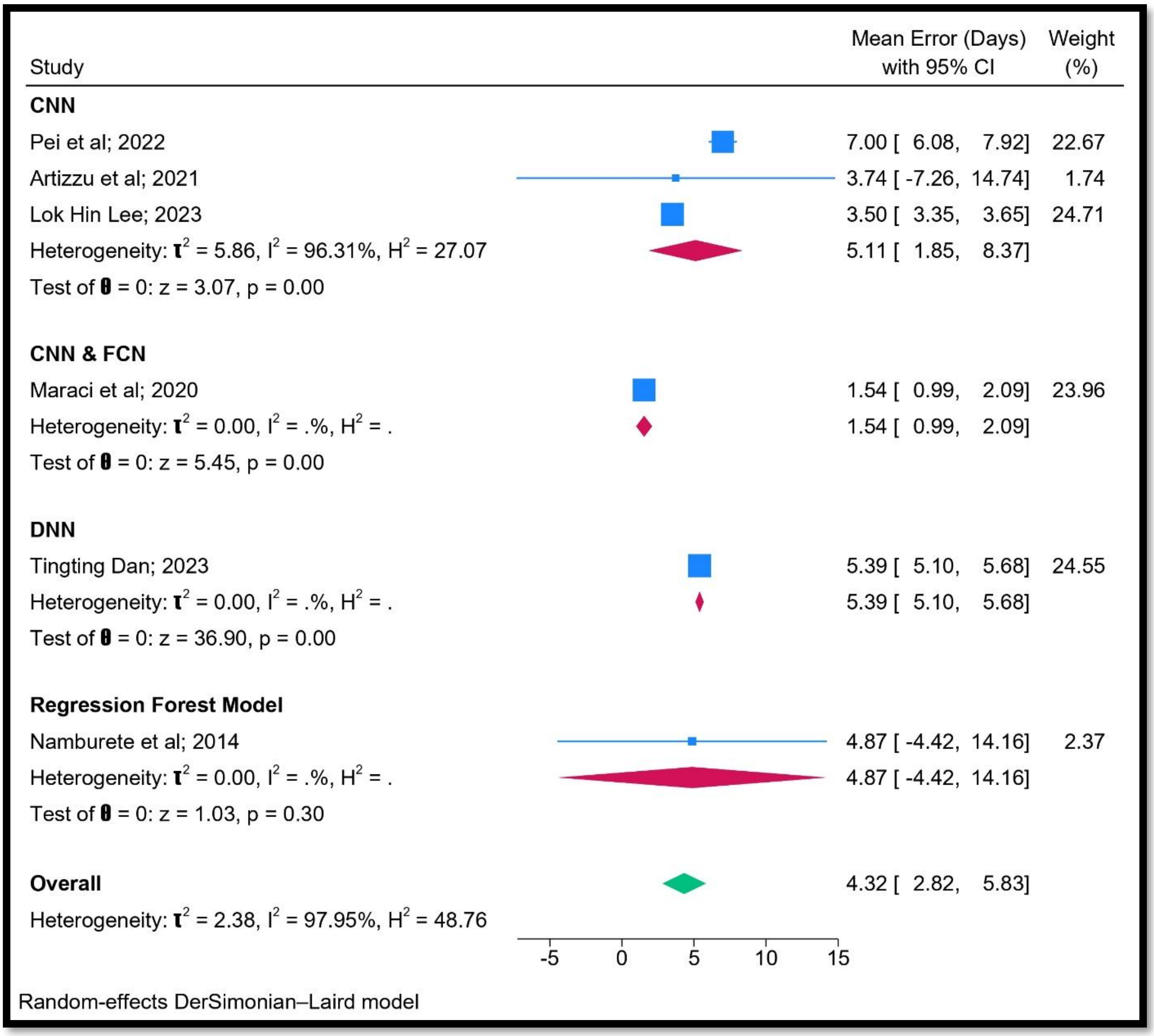

CNN= Convolutional Neural Network, FCN= Fully Convolutional Network, DNN= Deep Neural Network

Supplementary Figure 4: Forest plot of mean errors in GA estimation based on dataset validation

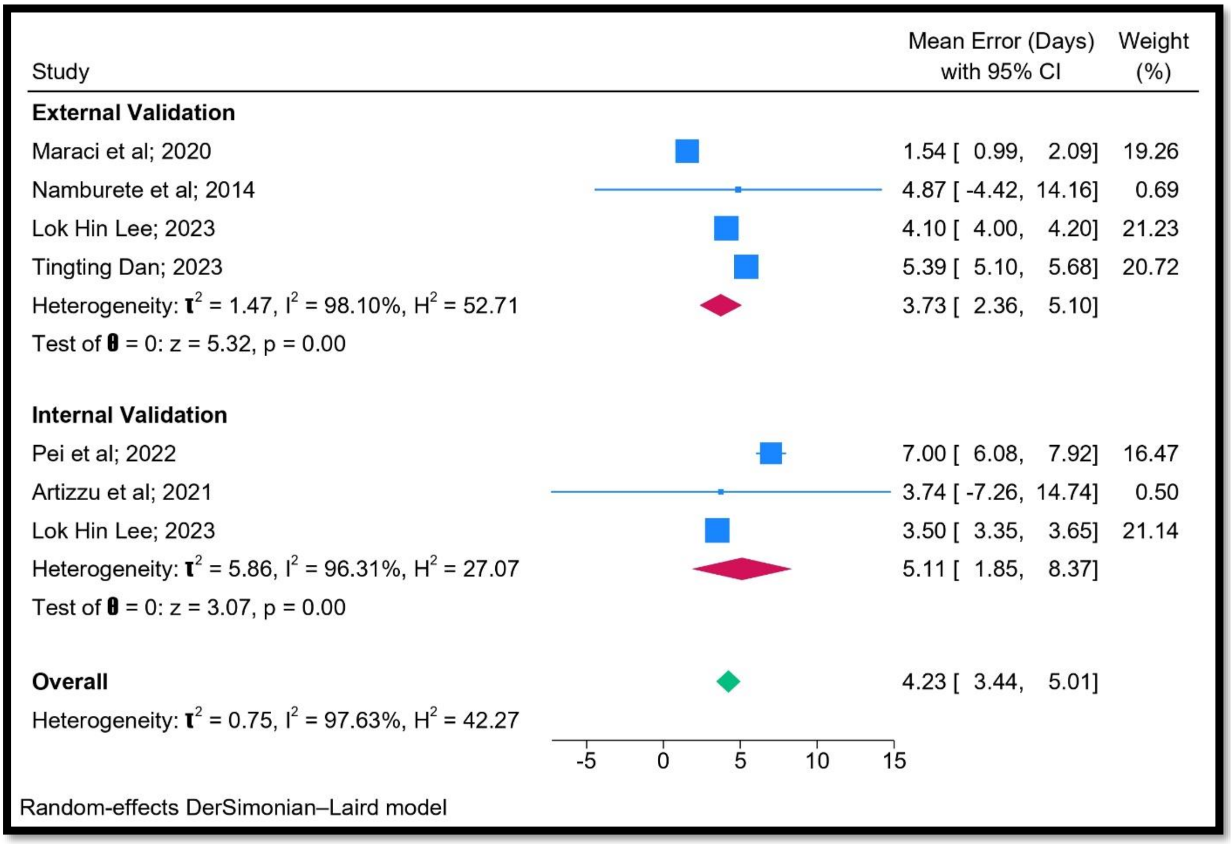

Lok Hin Lee; 2023 performed external and internal validation

Supplementary Figure 5: Forest plot of mean errors in GA estimation based on study designs

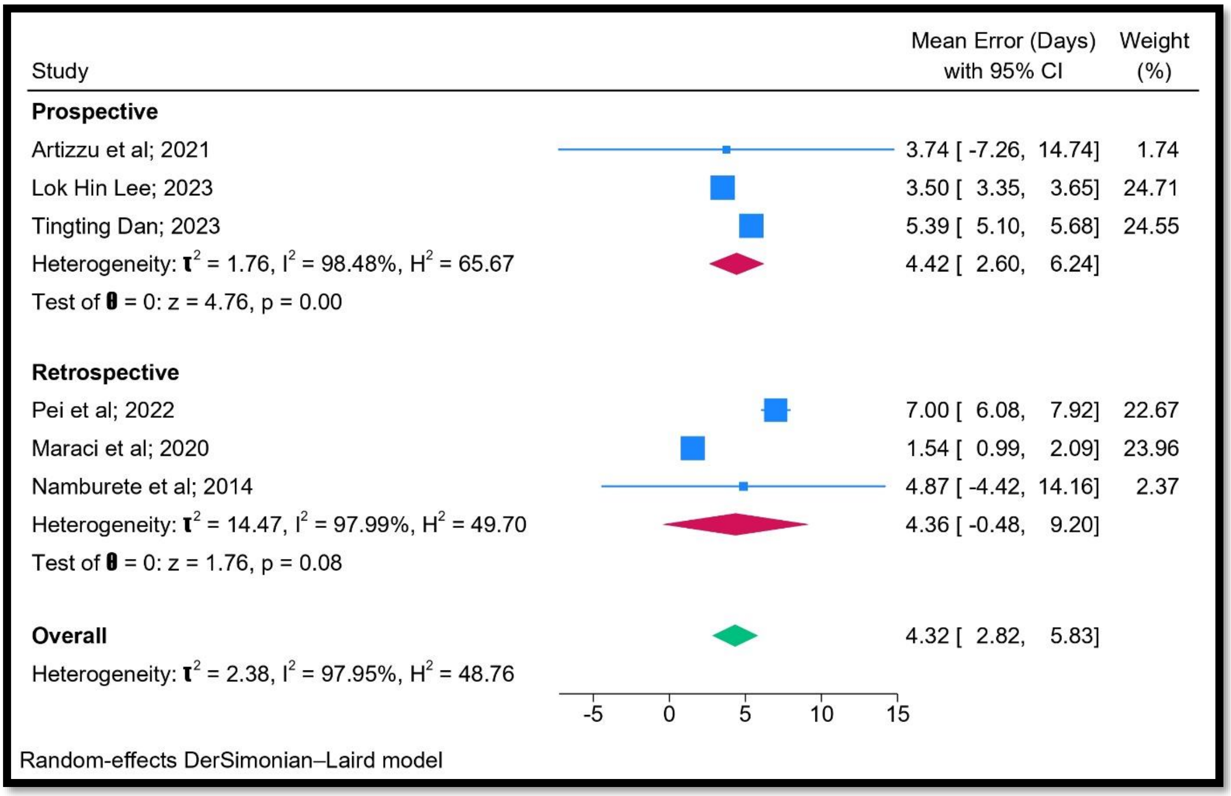

Supplement: Supplementary file 4 [file Datasheet4.pdf]
