# Supplementary material for: Use of artificial intelligence for gestational age estimation: a systematic review and meta-analysis
Source: Front Glob Womens Health. 2025 Jan 30;6:1447579. doi: 10.3389/fgwh.2025.1447579 (PMC11821921; doi:10.3389/fgwh.2025.1447579)
Supplement: Supplementary file 5 [file Datasheet5.pdf]

| **Use of artificial intelligence for gestational age estimation: A systematic review and meta-analysis** | |
|---|---|
| **Comments from Reviewer 4** | **Responses** |
| Comment1: Although this study conducts a comprehensive data analysis and reports the results of various subgroup analyses, it is recommended that the authors perform a sensitivity analyses by excluding studies with high risk of bias to further strengthen the reliability of the findings and ensure the broader applicability of the conclusions. | Thank you for your valuable feedback. We conducted a sensitivity analysis of blind sweep videos, excluding one study identified as having a high risk of bias. This analysis (n=3) yielded a pooled mean error of 2.62 days (95% CI: -0.22, 5.45; $I^2$ = 100%). We have incorporated this information on page 7, lines 163-164, and page 10, lines 233-235. Additionally, the corresponding forest plot has been included as Supplementary Figure 1. |
| Comment2: Although the study provides an estimate of the average error (3.56 days) in GA predictions by the AI model, it does not discuss the implications of these results for clinical decision-making in depth. It is recommended to further explore the potential impact of the AI model in reducing pregnancy delays or predicting preterm birth risk, especially in relation to different stages of pregnancy. | Thank you for your insightful comment. We have expanded the discussion to address the clinical implications of the AI model's performance on pages 12-13, lines 288-290. |
| Comment3: The training of AI models relies on high-quality image data, which is often of lower quality in LMICs. It is recommended to explore ways to overcome these challenges, such as using data augmentation or transfer learning techniques. | Thank you for your valuable comment. We have addressed this by adding that blind sweeps reduce the dependence on high-quality images, which can be challenging to obtain in low-resource settings on page 12, lines 281-283. |
| Comment4: Figure 1: There is a clear calculation error in the search flow diagram. | Thank you for highlighting the error. We corrected the calculation in the search flow diagram and revised Figure 1. |
